# Supplementary material for: Deamidation disrupts native and transient contacts to weaken the interaction between UBC13 and RING-finger E3 ligases
Source: eLife. 2019 Oct 22;8:e49223. doi: 10.7554/eLife.49223 (PMC6874479; doi:10.7554/eLife.49223)
Supplement: Supplementary file 1. [file elife-49223-supp1.docx]

**Supplementary Information**

**Deamidation disrupts native and transient contacts to weaken the interaction between UBC13 and RING-finger E3 ligases**

**Priyesh Mohanty^1^, Rashmi^1^, Batul Ismail Habibullah^1^, Arun G S^1^, and Ranabir Das^1,2^**

^1^National Centre for Biological Sciences, Tata Institute of Fundamental Research, Bengaluru-560065, India

**Table S1:** **Salt-bridge occupancies from conventional MD (200 ns) of UBC13/dUBC13 complexes.**

| **Salt-bridge** | **UBC13/TRAF6^RING^ (%)** | **dUBC13/TRAF6^RING^ (%)** |
| --- | --- | --- |
| R14 - E69 | 98.8 | 40.0 |
| R14 - E100 | - | 28.9 |

**Table S2. ΔG_PMF_ values reported for the UBC13/TRAF6^RING^ and the Barnase/Barstar complex.** Values are determined at 1.5 ns time intervals ranging from 2.5 to 10 ns.

| **ΔG_PMF_ (kcal mol^-1^)** | | | | | |  |
| --- | --- | --- | --- | --- | --- | --- |
| **Time interval (ns)** | **UBC13** | **dUBC13** | **R14A - UBC13** | **Q100A - UBC13** | **Bs - Br** |  |
| 2.5 - 4.0 | -2.26 | -0.24 | 0.78 | -0.60 | -12.5 |  |
| 4.0 - 5.5 | -1.96 | -1.18 | -0.37 | -0.37 | -15.1 |  |
| 5.5 - 7.0 | -2.48 | 0.86 | 1.11 | -2.12 | -12.8 |  |
| 7.0 - 8.5 | -0.99 | -0.89 | -0.94 | -2.90 | -10.5 |  |
| 8.5 - 10.0 | -2.45 | -2.03 | -0.37 | -2.19 | -11.8 |  |

**Table S3.** **F_max_ and unbinding work obtained from SMD trajectories of the UBC13/TRAF6^RING^ and dUBC13/TRAF6^RING^ complex**.

|  | **UBC13/TRAF6^RING^** | | **dUBC13/TRAF6^RING^** | |
| --- | --- | --- | --- | --- |
| **Trajectory No.** | **F_max_**  **(pN)** | **Work**  **(kcal mol^-1^)** | **F_max_**  **(pN)** | **Work**  **(kcal mol^-1^)** |
| 1 | 666.9 | 38.6 | 659.2 | 27.3 |
| 2 | 698.1 | 39.5 | 612.4 | 34.6 |
| 3 | 615.7 | 44.9 | 658.8 | 25.7 |
| 4 | 585.1 | 41.3 | 513.6 | 24.7 |
| 5 | 714.1 | 43.1 | 503.3 | 22.4 |
| 6 | 642.9 | 30.4 | 518.6 | 34.0 |
| 7 | 644.5 | 32.0 | 683.6 | 38.0 |
| 8 | 628.3 | 28.4 | 592.7 | 28.9 |
| 9 | 558.0 | 32.1 | 574.6 | 27.9 |
| 10 | 808.7 | 49.8 | 529.9 | 32.4 |

**Table S4.** **Salt-bridge occupancies in the SMD trajectories from 5 ns - 15 ns of the UBC13/TRAF6^RING^ and dUBC13/TRAF6^RING^ complex**.

|  | **UBC13/TRAF6^RING^ (%)** | **dUBC13/TRAF6^RING^ (%)** | |
| --- | --- | --- | --- |
| **Trajectory No.** | **R14 - E69** | **R14 - E69** | **R14 - E100** |
| 1 | 28.5 | 48.0 | 0.0 |
| 2 | 38.8 | 54.5 | 84.8 |
| 3 | 50.8 | 53.0 | 37.8 |
| 4 | 57.8 | 9.0 | 40.0 |
| 5 | 5.5 | 0.0 | 89.0 |
| 6 | 10.5 | 1.5 | 1.5 |
| 7 | 90.8 | 37.5 | 7.5 |
| 8 | 45.3 | 51.5 | 30.2 |
| 9 | 74.0 | 17.3 | 82.0 |
| 10 | 77.3 | 34.8 | 33.3 |

**Table S5. Salt-bridge occupancies from US windows.**

| **Salt-bridge** | **UBC13/TRAF6^RING^ (%)** | **dUBC13/TRAF6^RING^ (%)** |
| --- | --- | --- |
| R14 - E69 (2.7 nm) | 100.0 | 37.0 |
| R14 - E100 (2.7 nm) | - | 55.0 |
| R14 - D57 (3.0 nm) | 32.0 | 1.0 |
| R14 - E100 (3.0 nm) | - | 74.0 |

**Table S6 Salt-bridge occupancies from the two unbiased association trajectories that include native complexes.**

| **Salt-bridge** | **UBC13**  (%) | **dUBC13**  **(%)** |
| --- | --- | --- |
| R14 - E69 | 34.5 | 0.7 |
| R14 - E100 | - | 86.0 |
